# Factors Associated with Successful Smoking Cessation Among Adults in Saudi Arabia—A Cross-Sectional Study

**DOI:** 10.3390/healthcare13151813

**Published:** 2025-07-25

**Authors:** Nada A. Alyousefi, Reema S. Alquraini, Lina F. Alyahya, Norah M. Bin Hamad, Deema K. Aljuribah, Kadi K. Aldossari

**Affiliations:** 1 Department of Family and Community Medicine, College of Medicine, King Saud University, P.O. Box 2925, Riyadh 11461, Saudi Arabia; 2 College of Medicine, King Saud University (KSU), P.O. Box 2925, Riyadh 11461, Saudi Arabia; 442201278@student.ksu.edu.sa (R.S.A.); 442201276@student.ksu.edu.sa (L.F.A.); 442202180@student.ksu.edu.sa (N.M.B.H.); 442200797@student.ksu.edu.sa (D.K.A.); 442200778@student.ksu.edu.sa (K.K.A.)

**Keywords:** smoking cessation, motivations, public health, health behavior, motivation, Saudi Arabia

## Abstract

**Purpose:** Smoking is a major preventable cause of illness and death. Quitting smoking can reduce related health complications. Numerous factors, including age, socioeconomic status, smoking habits, and availability of support, influence smoking cessation success. Despite anti-smoking measures and smoking cessation clinics in Saudi Arabia, smoking-related deaths are increasing. This study aimed to identify factors influencing successful smoking cessation among Saudi adults and examine the relationship between cessation methods and success rates. **Patients and methods:** A cross-sectional study was conducted through an online survey targeting Saudi adults who had attempted to quit smoking of all types, whether traditional cigarettes, e-cigarettes, shisha, or others. Success was defined as quitting for at least six months. The data collected included sociodemographic details, smoking history, and experiences with cessation. Logistic regression analysis was used to identify factors associated with successful cessation. **Results:** Of 364 participants, 18.4% were successful quitters, with a mean age of 34.94. Occupation was a key predictor; freelance workers had significantly higher odds of unsuccessful quitting (OR = 12.96, 95% CI: 2.08–80.79, *p* = 0.006). Those who continued smoking despite illness were less likely to quit successfully (OR = 2.33, 95% CI: 1.16–4.68, *p* = 0.018). Early initiation of smoking also negatively impacted cessation success (OR = 1.10, 95% CI: 1.03–1.17, *p* = 0.006). Successful quitters reported fewer adverse symptoms during their attempts (*p* = 0.018) and employed behavioral strategies, such as disposing of tobacco products (*p* < 0.001), avoiding smoking triggers (*p* = 0.002), and engaging in exercise (*p* < 0.001). Confidence in quitting significantly contributed to success (*p* < 0.001). **Conclusions:** This study highlights the role of individual, social, and environmental factors in smoking cessation. Tailored interventions that address socioeconomic, psychological, and lifestyle factors are crucial for enhancing cessation success among Saudi adults.

## 1. Introduction

Smoking is a significant health risk factor that decreases an individual’s physical performance and quality of life [1]. Every year, 6 million people die from direct tobacco use and second-hand smoke exposure [1]. Despite growing awareness, smoking remains a leading preventable cause of disease and death both globally and in Saudi Arabia.

According to the 2019 Global Adult Tobacco Survey (GATS), 17.9% of the overall population in Saudi Arabia currently use tobacco, with a significantly higher prevalence among men (27.5%) compared to women (3.7%) [2].

Studies have shown a rising trend in deaths caused by smoking-related diseases in Saudi Arabia, with approximately 70,000 people losing their lives each year due to these conditions [2]. This high burden has prompted national surveys, such as the GATS, which in 2019 found that 19.8% of individuals currently use tobacco [3]. Approximately 37.9% attempted cessation in the past year, and 42.5% expressed an intention to quit within the next 12 months [3].

Successful smoking cessation, however, remains a challenge. A local survey conducted in 2013 revealed that although 51.3% of smokers attempted to quit within 12 months, only 25.3% were successful [4]. Research highlights several influencing factors, including age, gender, marital status, parenthood, occupation, health status, and individual motivation [5,6,7,8].

For example, older individuals and males tend to achieve higher success rates [5,6,7], while social factors, such as being married or a parent, have a positive influence on cessation [5]. Socioeconomic status also plays a role; unemployed individuals report lower success rates [5]. Health concerns, especially when reinforced by medical advice on cardiovascular or chronic conditions, have been shown to influence cessation decisions significantly [5,9]. Targeted interventions, such as diabetes-specific counseling, have demonstrated improved outcomes in randomized controlled trials [10].

Psychological factors, such as self-efficacy and the method of quitting (e.g., cold turkey versus assisted cessation), also influence success [5,8,11]. Although unassisted quitting is common, many lack access to support such as counseling or medications within primary care [12]. To address this, the Saudi Ministry of Health (MOH) has expanded cessation services, establishing 574 fixed and 98 mobile smoking cessation clinics (SCCs) nationwide [13,14]. Recent findings indicate that 68.6% of adult smokers and recent quitters attending SCCs attempted to quit, and 45.8% attempted to reduce their cigarette consumption [15]. Policy measures, including bans on smoking in public areas and increased taxation, have also contributed to behavioral change [5,15,16,17].

The use of different nicotine products, such as cigarettes, e-cigarettes, and shisha, remains widespread in Saudi Arabia despite ongoing efforts. Additionally, the cessation success rate remains suboptimal. Many contributing factors have been investigated, but no local data have assessed how these factors influence cessation outcomes among Saudi adults. This study aimed to identify factors that influence successful smoking cessation among Saudi adults. Understanding these factors can help increase quit rates, reduce smoking prevalence, and ultimately improve individual health outcomes and the overall health of society.

## 2. Materials and Methods

### 2.1. Study Design, Setting, and Participants

This study employed a cross-sectional design among Saudi adults who attempted to quit smoking to explore their smoking cessation experiences. The study was conducted in five regions of Saudi Arabia (Central region, West region, East region, South region, and North region). A convenience sampling technique was employed.

The questionnaire was distributed to both genders through various social media platforms. Participant eligibility was determined based on predefined inclusion criteria. The study participants were Saudi adults who were daily smokers (at least one cigarette per day) at some point in their lives and had attempted to quit smoking at least once. Based on three criteria: (1) They had smoked at least 100 cigarettes in their lives, (2) were former regular smokers, and (3) had attempted to quit smoking at least once. Participants who answered ‘no’ to these criteria were ineligible for the study. Those who responded ‘yes’ to all three questions were asked an additional question on their smoking behaviors. Those who currently smoked were classified as ‘unsuccessful quitters’ while those who had quit smoking for at least six months were identified as ‘successful quitters.’ Data for this study were collected between February 2024 and April 2024. To ensure geographic representation and to reach both smokers and ex-smokers, we recruited data collectors from all five regions of Saudi Arabia. Additionally, we collaborated with smoking cessation clinics in those provinces, which helped disseminate the survey among their attendees.

### 2.2. Measures and Data Collection

The outcome of this study was based on participants’ most recent smoking cessation attempt. Individuals who had successfully quit smoking for at least six months were classified as successful quitters, while those who had quit for less than six months were considered unsuccessful quitters. Various independent variables were measured, which included sociodemographic data, smoking history, smoking behaviors, smoking products, motivation for quitting, smoking cessation experiences of the last quitting attempt, which included the cessation method that has been used, the quitter’s behaviors during the cessation attempt period, withdrawal symptoms, techniques supporting quitting and preventing relapse, and other influencing factors such as the taxation.

The Arabic questionnaire utilized in this study was adapted from the validated English version used by a Thai study [5], with explicit authorization from the original corresponding author. The original questionnaire was translated into Arabic using a standard forward–backward translation protocol. A panel of bilingual experts, including two certified translators and two academic consultants in family medicine, reviewed both the forward and backward translations to ensure accuracy and cultural relevance. Discrepancies were discussed and resolved by consensus. Face and content validity were further assessed by two academic experts in family medicine and research methodology. Based on their feedback, minor refinements were made to enhance clarity and contextual appropriateness.

Additionally, the instrument incorporated the six items of the Fagerström Test for Nicotine Dependence to further assess smoking behaviors, which were available as open access for academic and research purposes, to assess individual indicators of nicotine dependence (time to first cigarette after waking; difficulty refraining in forbidden places; most difficult cigarette to give up; number of cigarettes per day; smoking frequency in first waking hours; smoking when ill) [18]. We did not calculate a composite score for this analysis. Instead, we report the distribution of responses for each item to highlight specific dependence behaviors.

The survey also included supplementary items derived from an extensive literature review to capture context-specific regulatory factors in Saudi Arabia, such as policies prohibiting smoking in public spaces and workplaces, as well as other relevant contextual influences on smoking cessation [7,8,10,15,19,20,21]. To establish content validity and cultural appropriateness, the finalized Arabic questionnaire was pre-tested and piloted among ten successful quitters and ten unsuccessful quitters, and it was reviewed by two experts who are family medicine consultants and academic researchers. Minor linguistic adjustments were made accordingly.

The questionnaire includes six sections and 43 questions. Since the survey link was shared via open social media platforms, it was not possible to track how many individuals received or viewed the invitation; therefore, the response rate could not be calculated.

### 2.3. Sample Size Calculation

The sample size was calculated using the single proportion formula, based on data from the Global Adult Tobacco Survey (GATS), published by the Centers for Disease Control and Prevention (CDC) in 2019. The survey reported that 37.9% of smokers had made a quit attempt in the past 12 months [3].

$$\frac{\left(1.96)2(0.379)(1-0.379\right)}{(0.05)2}\;=\;361.66$$

### 2.4. Data Analysis

Statistical analysis of the data was performed using IBM SPSS Statistical software for Windows, version 26.0 (IBM Corp., Armonk, NY, USA). Numerical data were presented as the mean and standard deviation (SD) and analyzed using an unpaired *t*-test. Categorical data were presented as frequencies and percentages and analyzed using the Chi-square test or exact test, as appropriate. Logistic regression analysis was performed to identify factors associated with unsuccessful quitting attempts. A multi-variable logistic regression analysis was conducted using the backward elimination method to determine the most significant predictors of unsuccessful smoking cessation. Analyzed data presented in the tables shows the association between smoking history and cessation methods used by successful and unsuccessful quitters by multiple characteristics such as smoking behaviors (regular, occasional), tobacco product types used (cigarette, E-cigarettes, and others), and whether having a chronic disease (yes, no), as contributors to a successful smoking cessation outcome. A set of factors that included motivation, other influencing factors on quitting smoking successfully, withdrawal symptoms, techniques supporting quitting and preventing relapse, and other factors on quitting smoking were presented as frequency and percentage. A significance level α = 0.05 was assumed, and 95% confidence intervals are used to report the statistical significance and precision of the results.

## 3. Results

A total of 364 participants were included in this study, with a mean age of 33.1 ± 10.94 years. The sample consisted of a male predominance, representing 89.6% of the participants, as shown in Table 1. In terms of current smoking status, 70.9% were smokers, while 10.7% stopped less than 6 months ago and 18.4% stopped more than 6 months ago; there was a statistically significant relation between quitting status and occupation (*p* < 0.001). Retired individuals represent a higher proportion among successful quitters (14.9%) than unsuccessful quitters (2.4%). Private sector employees exhibit a lower success rate, comprising 29.3% of unsuccessful quitters compared to 17.9% of successful quitters. A trend-level association was also observed between education and quitting status (*p* = 0.053), which may suggest a potential relationship worth further investigation. There was also a significant difference between the two groups regarding the age at which they first smoked cigarettes (years), and the age at which they began to smoke every day (years). Successful quitters began smoking at a younger age (mean age: 17.95 ± 5.41 years) compared to unsuccessful quitters (19.91 ± 5.69 years), with a statistically significant difference (*p*-value = 0.012). Successful quitters also transitioned to daily smoking earlier (20.25 ± 5.24 years) compared to unsuccessful quitters (22.17 ± 6.10 years), (*p*-value = 0.019).

The rate of smoking while sick was significantly higher among unsuccessful quitters than the successful ones (37% vs. 23.9%, *p* = 0.041) as shown in Table 2.

Table 3 shows several variables that demonstrate significant associations with quitting status. Economic reasons, such as increased tobacco prices and taxation, were more commonly cited as motivations among unsuccessful quitters (25.6%) compared to successful quitters (13.4%), indicating that financial factors may strongly influence initial cessation attempts (*p* = 0.034). Successful quitters were more likely to report no adverse symptoms during their cessation attempts (22.4% vs. 11.4%, *p* = 0.018), suggesting a smoother transition for some. Endurance and persistence were significantly higher among successful quitters (58.2% vs. 40.1%, *p* = 0.007). Behavioral strategies also varied, with successful quitters being more likely to dispose of tobacco products (44.8% vs. 21.9%, *p* < 0.001), avoid social triggers such as friends who smoke (38.8% vs. 21.2%, *p* = 0.002), and engage in exercise (35.8% vs. 16.2%, *p* < 0.001), emphasizing the importance of environmental modifications and lifestyle changes. Additionally, successful quitters had greater confidence in their ability to quit (68.7% vs. 41.1%, *p* < 0.001). While the increase in cigarette value-added tax influenced cessation attempts more among unsuccessful quitters (49.2% vs. 31.3%, *p* = 0.008), it was insufficient to ensure success.

Table 4 presents the results of a multivariable logistic regression analysis, conducted using the backward elimination method, to identify the most significant predictors of unsuccessful smoking cessation. The results of the multiple logistic regression identified several factors significantly associated with unsuccessful smoking cessation. Occupation was a prominent predictor, with freelance laborers having the highest odds of unsuccessful quitting compared to retired individuals (adjusted OR = 12.96, 95% CI: 2.08–80.79, *p* = 0.006). Similarly, private sector employees (adjusted OR = 7.28, 95% CI: 2.14–24.78, *p* = 0.002), government officials (adjusted OR = 3.84, 95% CI: 1.22–12.12, *p* = 0.022), unemployed participants (adjusted OR = 5.21, 95% CI: 1.01–26.91, *p* = 0.049), and students (adjusted OR = 6.8, 95% CI: 1.98–23.32, *p* = 0.002) also exhibited higher odds of unsuccessful quitting compared to retired individuals. The age at which participants first smoked cigarettes was another significant factor, with each additional year associated with a higher likelihood of unsuccessful quitting (adjusted OR = 1.1, 95% CI: 1.03–1.17, *p* = 0.006). Smoking, even when sick in bed, was associated with increased odds of unsuccessful cessation (adjusted OR = 2.33, 95% CI: 1.16–4.68, *p* = 0.018). The duration for which cessation-related symptoms disturbed daily life also played a significant role. Participants who experienced symptom disturbances for 2–3 weeks (adjusted OR = 0.21, 95% CI: 0.09–0.53, *p* < 0.001) or more than one month (adjusted OR = 0.23, 95% CI: 0.09–0.6, *p* = 0.002) were less likely to unsuccessfully quit smoking compared to those disturbed for about one week. This finding is counterintuitive and may reflect a statistical artifact; therefore, it should be interpreted with caution.

## 4. Discussion

This study identifies key factors that influence the success of smoking cessation among Saudi adults who have quit for at least six months and emphasizes the need to address socioeconomic, psychological, and lifestyle factors to improve smoking cessation outcomes in this population.

Socioeconomic factors play an important role in smoking behavior. For example, age may influence smoking cessation outcomes. In this study, successful quitters had a mean age of approximately 35 years. Younger individuals generally find it easier to quit due to fewer years of habitual smoking and possibly a higher chance of being influenced by other motivations. However, in a study conducted in Thailand, successful quitters had a mean age of 62, whereas a Korean study found that the highest incidence of successful quitters is observed in those aged 65 and above [5,8]. Education levels can correlate with access to resources and awareness of the health risks associated with smoking, potentially impacting cessation success rates. The current study confirmed this, i.e., college education was associated with successful quitting.

Occupation was a significant predictor, showing that freelance workers had higher odds of unsuccessful quitting. This can be explained by the fact that workplace culture and stress levels can either facilitate or hinder quitting efforts. Similarly, individuals with a monthly income between SAR 5000 and 15,000 were more likely to quit smoking successfully. These findings were consistent with the previous literature [5,22]. Notably, place of residence, marital status, having children, and the presence of chronic diseases did not significantly influence the success of quitting.

Exploring the smoking history and cessation methods as possible influences for success in quitting. The mean age of initiation of smoking and the mean age of regular smoking show a significant difference between successful quitters and unsuccessful quitters, in which successful quitters began smoking earlier compared to unsuccessful quitters; also, successful quitters transitioned to daily smoking earlier compared to unsuccessful quitters. Several differences have been found concerning smoking habits and patterns among unsuccessful and successful quitters. Successful quitters were less likely to experience difficulty refraining from smoking in forbidden places, despite a similar proportion of both groups finding the first cigarette of the morning hardest to give up. While the frequency of smoking (daily vs. intermittent) did not significantly differ from each other, successful quitters were less likely to smoke even when sick in bed. Interestingly, the highest proportion of successful and unsuccessful quitters primarily used only cigarettes.

This study found that successful quitters were more likely to report no adverse symptoms during their cessation attempts, suggesting a smoother transition for some. This study revealed that severe cravings were prevalent among participants, a finding similar to those of Thailand’s study [5]. Additionally, symptoms of depression and difficulty sleeping were experienced, similar to the findings of Thailand’s study [5]. These were the most common nicotine withdrawal symptoms observed in our participants. Our study found that quitters who had a good appetite after quitting were more likely to succeed in smoking cessation; however, this could potentially result in weight gain, as reported by participants. However, increased appetite and weight gain suggest the importance of addressing psychosocial factors in cessation interventions [6].

Addressing motivational factors like stopping smoking for religious reasons, medical problems, or family reasons like a child or spouse has heavier impact on a successful quitting attempt than quit attempts motivated by a doctor, nurse, health professional, or economic reasons. This study found that self-confidence was a predictor of successful quit attempts. Endurance and persistence were significantly higher among successful quitters, highlighting the role of self-efficacy in the cessation process.

This study also emphasizes the importance of environmental modifications and lifestyle changes, which align with other studies [5,6]. Successful quitters tend to adopt specific behavioral strategies, such as discarding tobacco products and distancing themselves from social environments that may trigger relapse, like spending time with friends who smoke. Many also incorporate regular physical activity into their routines. Chronic nicotine exposure alters the mesocorticolimbic dopamine pathway, which plays a key role in processing rewarding stimuli; as a result, the brain becomes dependent on nicotine to maintain feelings of reward and to avoid withdrawal [5,23]. Aerobic exercise, which naturally increases dopamine and serotonin levels, has been shown to enhance mood and is recognized as a positive predictor of smoking cessation success [24].

Interestingly, most successful quitters did not use medication, contrary to the outcomes of a Turkish study and Alwhaibi’s study, which suggest the need for further investigation into the effectiveness of pharmacological interventions [19,25]. Most successful quitters reported that they heard about SCC in Saudi Arabia, but did not benefit from its services. Utilization of smoking cessation clinics was low among both successful and unsuccessful quitters, indicating a need for improved access to cessation resources, which is similar to Alwhaibi’s study [25]. Despite awareness of smoking cessation services and the impact of cigarette prices, these factors did not significantly affect cessation outcomes in the current study, possibly due to limitations in the sample size. However, value-added tax significantly did not affect successful smoking cessation [14,15,17]. While the increase in cigarette value-added tax influenced cessation attempts more among unsuccessful quitters (49.2% vs. 31.3%, *p* = 0.008), it was insufficient to ensure success. It was found that an increase in cigarette prices leads to a reduction in smoking rate in Saudi Arabia [15,21].

### Strength and Limitation

While this study provides valuable insights into the factors influencing smoking cessation among Saudi adults through a detailed questionnaire to study the smoking history to assess the impact of it on the quitting experience, assessing multiple motivations at the personal level and environment of the smoker, the techniques support quitting and prevent relapse, several limitations need to be addressed. First, the number of participants was relatively small, which made it difficult to analyze the effects of potentially interesting factors. Recruiting more participants will help obtain more comprehensive results to assess the overall effect of the multiple factors on smoking cessation attempts nationwide. The use of convenience sampling limits the generalizability of findings. Participants were recruited through an online survey, which likely attracted more motivated individuals with internet access. This self-selection bias suggests that the results may not accurately reflect the experiences of all smokers in Saudi Arabia, particularly those who are less engaged or lack internet access. Second, there is a possibility of recall bias among the participants, considering the nature of the cross-sectional study. Participants relied on their memories of past quit attempts, which may have occurred months or years ago. This can lead to inaccuracies in reporting motivations and behaviors, as individuals may struggle to recall specific details. Current feelings may also influence how past experiences are remembered. We included the six items of the Fagerström Test for Nicotine Dependence to highlight specific dependence behaviors, but we did not compute the overall score, which may limit direct comparison with studies using the total dependence index. Although the Arabic version of the questionnaire underwent expert review and pilot testing to ensure clarity and relevance, formal psychometric validation (e.g., internal consistency or construct validity) was not conducted. This was due to the exploratory nature of the study and the focus on descriptive insights rather than the development or testing of a new measurement scale. Future studies may consider performing a full-scale psychometric evaluation of the Arabic version. Also, the cross-sectional nature of this study limits the ability to establish causality. While associations between factors like confidence and successful quitting were identified, it remains unclear whether increased confidence leads to quitting or if successful quitting enhances confidence. This highlights the need for longitudinal studies to clarify causal relationships. Moreover, exploring potentially confounding factors affecting smoking cessation would benefit from a qualitative approach.

## 5. Conclusions

In conclusion, this study sheds light on the complex interplay between individual, social, and environmental factors that influence smoking cessation among Saudi adults. The findings underscore the importance of tailored cessation interventions that address socioeconomic disparities, psychosocial factors, and individual motivations. By understanding the diverse range of influences on cessation outcomes, policymakers and healthcare providers can develop more effective smoking cessation programs that cater to the unique needs of individuals attempting to quit.

## Figures and Tables

**Table 1:** Relation between sociodemographic characteristics of participants and quitting status.

Item	Unsuccessful Quitters (n = 297)	Successful Quitters (n = 67)	*p*-Value
**Age (years)**	32.65 ± 10.30	34.94 ± 13.37	0.192
**Gender**			
Male	266 (89.6%)	60 (89.6%)	0.998
Female	31 (10.4%)	7 (10.4%)	
**Education**			
Secondary education or less	46 (15.5%)	10 (14.9%)	0.053
College	216 (72.7%)	43 (64.2%)	
Postgraduate	33 (11.1%)	11 (16.4%)	
Other	2 (0.7%)	3 (4.5%)	
**Occupation**			
Retired	7 (2.4%)	10 (14.9%)	<0.001
Freelance labor	25 (8.4%)	2 (3.0%)	
Private sector employee	87 (29.3%)	12 (17.9%)	
Government official	79 (26.6%)	24 (30.2%)	
Housewife	5 (1.7%)	2 (3.0%)	
Unemployed	21 (7.1%)	3 (4.5%)	
Student	73 (24.6%)	14 (20.9%)	
**Monthly Income (Saudi Riyal)**			
I do not have an income	42 (14.1%)	11 (16.4%)	0.209
Less than SAR 5 thousand	66 (22.2%)	11 (16.4%)	
SAR 5–15 thousand	104 (35.0%)	26 (38.8%)	
SAR 16–20 thousand	41 (13.8%)	15 (22.4%)	
SAR 21–30 thousand	24 (8.1%)	2 (3.0%)	
More than SAR 30 thousand	20 (6.7%)	2 (3.0%)	
**Place of residence**			
Eastern Region	35 (11.8%)	10 (14.9%)	0.254
Central Region	118 (39.7%)	34 (50.7%)	
Western Region	88 (29.6%)	14 (20.9%)	
Southern region	23 (7.7%)	2 (3.0%)	
Northern region	33 (11.1%)	7 (10.4%)	
**Marital status**			
Single	155 (52.2%)	29 (43.3%)	0.149
Married	127 (42.8%)	36 (53.7%)	
Divorced	14 (4.7%)	1 (1.5%)	
Widowed	1 (0.3%)	1 (1.5%)	
**Having children**			
No	179 (60.3%)	34 (50.7%)	0.153
Yes	118 (39.7%)	33 (49.3%)	
**Chronic diseases**			
No	219 (73.7%)	48 (71.6)	0.726
Yes	78 (26.3%)	19 (28.4	
Obesity	32 (10.8%)	7 (10.4%)	0.938
Hypertension	17 (5.7%)	4 (6.0%)	0.999
Diabetes	16 (5.4%)	5 (7.5%)	0.560
Asthma	11 (3.7%)	4 (6.0%)	0.492
Other	14 (4.7%)	2 (3.0%)	0.746
**Age at which participants first smoked cigarettes (years)**	19.91 ± 5.69	17.95± 5.41	0.012
**Age at which participants began to smoke every day (years)**	22.17 ± 6.10	20.25 ± 5.24	0.019

Note: Numerical data are presented as mean ± SD, and categorical data are presented as frequency (%), Statistical significance at *p*-value < 0.05. A significance level α = 0.05 was assumed.

**Table 2:** The relation between the smoking behavior of participants and quitting status.

Item	Unsuccessful Quitters (n = 297)	Successful Quitters (n = 67)	*p*-Value
**How soon after you wake up do you usually have your first smoke?**			
In 5 min	75 (25.3%)	15 (22.4%)	0.950
6–30 min	74 (24.9%)	17 (25.4%)	
31–60 min	55 (18.5%)	12 (17.9%)	
More than 60 min	93 (31.3%)	23 (34.3%)	
**Do you find it difficult to refrain from smoking in places where it is forbidden? Mosques, public spaces**			
No	183 (61.6%)	42 (62.7%)	0.871
Yes	114 (38.4%)	25 (37.3%)	
**Which cigarettes would you hate to give up?**			
The first in the morning	170 (57.2%)	35 (52.2%)	0.456
Any other	127 (42.8%)	32 (47.8%)	
**How many cigarettes a day do you smoke?**			
10 or less	88 (29.6%)	21 (31.3%)	0.342
11–20	135 (45.5%)	27 (40.3%)	
21–30	46 (15.5%)	8 (11.9%)	
31 or more	28 (9.4%)	11 (16.4%)	
**Do you smoke more frequently in the morning?**			
No	164 (55.2%)	45 (67.2%)	0.074
Yes	133 (44.8%)	22 (32.8%)	
**Do you smoke even if you are sick in bed most of the day?**			
No	187 (63%)	51 (76.1%)	0.041
Yes	110 (37%)	16 (23.9%)	
**Typically, what kind of tobacco products do you use?**			
Cigarette	220 (74.1%)	50 (74.6%)	0.093
E-cigarette	56 (18.9%)	7 (10.4%)	
Shisha	17 (5.7%)	8 (11.9%)	
Other	4 (1.3%)	2 (3.0%)	
**What is the second type of tobacco product you usually use?**			
None	2 (0.7%)	0 (0%)	0.240
Cigarette	127 (42.8%)	23 (34.3%)	
E-cigarette	82 (27.6%)	23 (34.3%)	
Shisha	76 (25.6%)	21 (31.3%)	
Other	10 (3.4%)	0 (0%)	
**How often do you smoke?**			
Regularly (every day)	240 (80.8%)	58 (86.6%)	0.269
Intermittent (not every day)	57 (19.2%)	9 (13.4%)	

Note: Numerical data are presented as mean ± SD, and categorical data are presented as frequency (%), Statistical significance at *p*-value < 0.05. A significance level α = 0.05 was assumed.

**Table 3:** Association between smoking cessation trials and quitting status.

Item	Unsuccessful Quitters	Successful Quitters	*p*-Value
	(n = 297)	(n = 67)	
**Did you quit smoking by yourself or by receiving some form of help (e.g., medicine or counseling)**			
I quit smoking by myself	226 (76.1%)	56 (83.6%)	0.185
I received some help (e.g., medicine or counseling)	71 (23.9%)	11 (16.4%)	
**What were the causes and motivations that made you decide to stop smoking?**			
For my child/grandchild	36 (12.1%)	6 (9%)	0.533
Family member (child or spouse)	57 (19.2%)	16 (23.9%)	0.4
Relative (parent, sibling, other relative)	71 (23.9%)	15 (22.4%)	0.792
Due to a medical problem, a doctor, nurse, or health professional asked you to stop smoking	44 (14.8%)	11 (16.4%)	0.741
A doctor, nurse, or health professional asked you to stop smoking, unrelated to a medical problem	26 (8.8%)	4 (6%)	0.454
A health volunteer asked me to stop smoking (this made me initiate a quit attempt)	31 (10.4%)	8 (11.9%)	0.719
Economic reasons (increase in tobacco price, taxation)	76 (25.6%)	9 (13.4%)	0.034
Religious reasons	80 (26.9%)	23 (34.3%)	0.225
Warning on tobacco packs	82 (27.6%)	13 (19.4%)	0.167
Other	46 (15.5%)	17 (25.4%)	0.053
**After you started your cessation attempt this time, what following adverse reactions did you experience?**			
Severe cravings	196 (66%)	36 (53.7%)	0.059
Irritability, hostility, and was easily stressed	128 (43.1%)	29 (43.3%)	0.978
Depressed	80 (26.9%)	13 (19.4%)	0.202
Headache, dizziness	85 (28.6%)	20 (29.9%)	0.841
Difficulty sleeping	45 (15.2%)	8 (11.9%)	0.501
Increase in appetite and weight gain	62 (20.9%)	22 (32.8%)	0.036
Constipation	9 (3.0%)	1 (1.5%)	0.696
Other	4 (1.3%)	0 (0%)	0.597
None of the above symptoms at all	34 (11.4%)	15 (22.4%)	0.018
**Overall, for how long did the symptoms you reported in the previous question disturb your daily life?**			
Did not disturb me at all	24 (8.1%)	6 (9%)	0.003
Disturbed me for about one week	112 (37.7%)	9 (13.4%)	
Disturbed me for about 2–3 weeks	46 (15.5%)	18 (26.9%)	
Disturbed me for about 1 month	47 (15.8%)	11 (16.4%)	
Disturbed me for more than 1 month	49 (16.5%)	16 (23.9%)	
None of the symptoms occurred	19 (6.4%)	7 (10.4%)	
**What additional method (s) did you use to help you stop smoking?**			
Avoided meeting friends and others who smoke	0 (0%)	1 (1.5%)	0.184
Use some things to reduce your acute desire (e.g., eating sour foods, using mouthwash, brushing teeth, eating candy, chewing gum, chewing a toothpick)	100 (33.7%)	15(22.4%)	0.073
Engage in leisure activities (e.g., watching TV, eating food)	75 (25.3%)	19 (28.4%)	0.6
Exercise	110 (37%)	30 (44.8%)	0.24
Work	57 (19.2%)	15 (22.4%)	0.553
Eat or drink herbs	16 (5.4%)	5 (7.5%)	0.56
General massage and relaxing exercise	11 (3.7%)	3 (4.5%)	>0.999
Foot massage	6 (2.0%)	1 (1.5%)	>0.999
Other	11 (3.7%)	8 (11.9%)	0.012
No additional method was used	89 (30%)	16 (23.9%)	0.321
**If you passed the initial stage of smoking cessation, what additional method (s) did you use to prevent a smoking relapse?**			
Endurance, persistence	119 (40.1%)	39 (58.2%)	0.007
Disposal of tobacco and related products	65 (21.9%)	30 (44.8%)	<0.001
Avoided meeting friends and others who smoke	63 (21.2%)	26 (38.8%)	0.002
Employing strategies to manage craving time during high craving times, including after meals, before going to sleep, or when stressed	24 (8.1%)	7 (10.4%)	0.531
Use some things to reduce your acute desire (e.g., eating sour foods, using mouthwash, brushing teeth, eating candy, chewing gum, chewing a toothpick)	24 (8.1%)	9 (13.4%)	0.168
Engage in leisure activities (e.g., watching TV, eating food)	35 (11.8%)	9 (13.4%)	0.709
Exercise	48 (16.2%)	24 (35.8%)	<0.001
Work	33 (11.1%)	9 (13.4%)	0.591
Eat or drink herbs	7 (2.4%)	5 (7.5%)	0.05
General massage and relaxing exercise	6 (2%)	3 (4.5%)	0.375
Other	2 (0.7%)	0 (0%)	>0.999
No additional method was used	43 (14.5%)	11 (16.4%)	0.687
**Which of the following did you encounter during your current attempt to quit smoking that made quitting easier?**			
I had confidence that I could successfully stop smoking	122 (41.1%)	46 (68.7%)	<0.001
My family encouraged and helped me stop smoking	67 (22.6%)	20 (29.9%)	0.206
We had a rule not to smoke at home	30 (10.1%)	3 (4.5%)	0.294
I saw a person who had successfully stopped smoking	57 (19.2%)	12 (17.9%)	0.809
Tobacco products were expensive	37 (12.5%)	5 (7.5%)	0.248
Smoking in public places was prohibited	14 (4.7%)	4 (6%)	0.754
Smoking in workplaces was prohibited	18 (6.1%)	4 (6%)	>0.999
I feel bad-smelling when I smoke again after quitting for a while	55 (18.5%)	14 (20.9%)	0.654
There were social reasons that helped me stop smoking	57 (19.2%)	13 (19.4%)	0.968
There were religious reasons that helped me stop smoking	54 (18.2%)	16 (23.9%)	0.285
None	55 (18.5%)	6 (9%)	0.085
**Did the price of cigarette products support you to quit smoking?**			
No	164 (55.2%)	42 (62.7%)	0.265
Yes	133 (44.8%)	25 (37.3%)	
**Did the increase in value-added tax on cigarette packs help you quit smoking?**			
No	151 (50.8%)	46 (68.7%)	0.008
Yes	146 (49.2%)	21 (31.3%)	
**Have you heard about smoking cessation clinics in Saudi Arabia?**			
No	57 (19.2%)	7 (10.4%)	0.089
Yes	240 (80.8%)	60 (89.6%)	
**Have you ever benefited from the services of the smoking cessation clinics in Saudi Arabia?**			
No	211 (71%)	54 (80.6%)	0.112
Yes	86 (29%)	13 (19.4%)	

Note: Categorical data are presented as frequency (%). A significance level α = 0.05 was assumed.

**Table 4:** Multiple logistic regression for factors associated with unsuccessful quitting of smoking.

	Univariate Analysis				Multivariable Analysis			
		95% Confidence Interval of OR				95% Confidence Interval of OR		
Factors	OR	Lower	Upper	*p*-Value	OR	Lower	Upper	*p*-Value
**Age**	0.98	0.96	1.00	0.124				
**Occupation:**								
Retired	1.00				1.00			
Freelance labor	17.86	3.15	101.2	0.001	12.96	2.08	80.79	0.006
Private sector employee	10.36	3.32	32.35	<0.001	7.28	2.14	24.78	0.002
Government official	4.70	1.62	13.69	0.005	3.84	1.22	12.12	0.022
Housewife	3.57	0.53	23.95	0.190	1.87	0.21	16.29	0.573
Unemployed	10.00	2.13	47.02	0.004	5.21	1.01	26.91	0.049
Student	7.45	2.42	22.89	<0.001	6.8	1.98	23.32	0.002
**Marital status:**								
Single	1.00							
Married	0.66	0.38	1.14	0.133				
Divorced	2.62	0.33	20.7	0.361				
Widowed	0.19	0.01	3.08	0.241				
**Having children (yes)**	0.68	0.4	1.16	0.154				
**Age at which participants first smoked cigarettes (years)**	1.08	1.02	1014	0.013	1.10	1.03	1.17	0.006
**Smoking more frequently in the morning (yes)**	1.66	0.95	2.9	0.076				
**Smoking, even if being sick in bed most of the day (yes)**	1.87	1.02	3.45	0.043	2.33	1.16	4.68	0.018
**The kind of tobacco products used**								
Cigarette	1.00							
E-cigarette	1.82	0.78	4.23	0.165				
Shisha	0.48	0.2	1.18	0.111				
Other	0.45	0.08	2.55	0.37				
**Severe cravings as a side effect when trying to stop smoking (yes)**	1.67	0.98	2.86	0.061				
**Duration of the symptoms disturbs daily life**								
Did not disturb at all	0.32	0.1	0.99	0.048	0.4	0.12	1.37	0.144
Disturbed for about one week	1.00				1.00			
Disturbed for about 2–3 weeks	0.21	0.09	0.49	<0.001	0.21	0.09	0.53	<0.001
Disturbed for about 1 month	0.34	0.13	0.88	0.027	0.37	0.14	1.02	0.054
Disturbed for more than 1 month	0.25	0.1	0.6	0.002	0.23	0.09	0.6	0.002
None of the symptoms occurred	0.22	0.07	0.66	0.007	0.4	0.12	1.34	0.138

Note. Estimates represent the log odds of “unsuccessful quitters” versus “successful quitters.” Statistical significance at *p*-value < 0.05. Abbreviation: Smoking cessation clinics (SCC), Ministry of Health (MOH).

## Data Availability

The datasets generated and analyzed during the current study are available from the corresponding author on reasonable request.
